# Interface Characteristics of Sapphire Direct Bonding for High-Temperature Applications

**DOI:** 10.3390/s17092080

**Published:** 2017-09-11

**Authors:** Wangwang Li, Ting Liang, Yulei Chen, Pinggang Jia, Jijun Xiong, Yingping Hong, Cheng Lei, Zong Yao, Lei Qi, Wenyi Liu

**Affiliations:** 1 Science and Technology on Electronic Test & Measurement Laboratory, North University of China, Taiyuan 030051, China; 18434365707@163.com (W.L.); cyl029@126.com (Y.C.); pgjia@cqu.edu.cn (P.J.); xiongjijun@nuc.edu.cn (J.X.); hongyingping@nuc.edu.cn (Y.H.); leichengnuc@163.com (C.L.); 2 Key Laboratory of Instrumentation Science & Dynamic Measurement, Ministry of Education, North University of China, Taiyuan 030051, China; 3 North Automatic Control Technology Institute, Taiyuan 030006, China; yaozong126@sina.com (Z.Y.); qilei19850224@163.com (L.Q.)

**Keywords:** sapphire, direct bonding, vacuum-sealed cavity, pressure sensor, high temperature

## Abstract

In this letter, we present a sapphire direct bonding method using plasma surface activation, hydrophilic pre-bonding, and high temperature annealing. Through the combination of sapphire inductively coupled plasma etching and the direct bonding process, a vacuum-sealed cavity employable for high temperature applications is achieved. Cross-sectional scanning electron microscopy (SEM) research of the bonding interface indicates that the two sapphire pieces are well bonded and the cavity structure stays intact. Moreover, the tensile testing shows that the bonding strength of the bonding interface is in excess of 7.2 MPa. The advantage of sapphire direct bonding is that it is free from the various problems caused by the mismatch in the coefficients of thermal expansion between different materials. Therefore, the bonded vacuum-sealed cavity can be potentially further developed into an all-sapphire pressure sensor for high temperature applications.

## 1. Introduction

There is no measurement technology currently available to achieve direct, accurate measurements of static and dynamic pressure changes in extremely high temperature environments such as gas turbines, high-speed combustors, and other aerospace propulsion applications [1]. Conventional silicon pressure sensors and even silicon-on-insulator (SOI) sensors cannot operate at high temperatures over 500 °C due to the plastic deformation of the material [2,3]. In addition, some micromachined pressure sensors based on silicon carbide [4,5,6] and ceramic [7,8] have also been developed. However, these sensors also have significant limitations on operating at extremely high temperatures over 1000 °C due to the degradation of mechanical and electrical properties. Sapphire, with its characteristics of a high melting point of 2040 °C, resistance to chemical corrosion, high mechanical strength, and excellent optical qualities, has been considered an ideal material for high temperature sensing applications. Compared with the sapphire pressure sensors based on electrical detection mechanism [9], sapphire fiber optic pressure sensors have attracted considerable attention due to their advantages of being immune to electromagnetic interference and non-conductive properties [10,11,12]. An all-sapphire pressure sensor has the potential to achieve accurate pressure measurements at extremely high temperatures over 1500 °C [13]. The core technical challenge for the development of all-sapphire pressure sensors based on extrinsic Fabry–Perot interferometer (EFPI) is the hermetic bonding for the sensor cavity.

**Figure 1:**
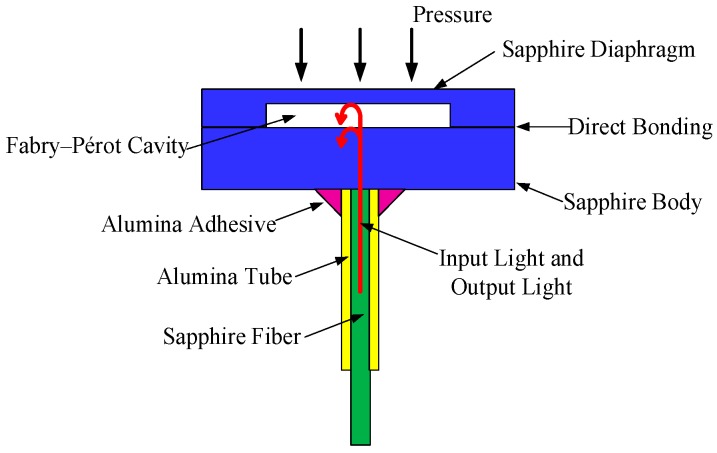
Structure schematic of the all-sapphire pressure sensor.

Sapphire direct bonding refers to the phenomenon wherein two smooth and flat sapphire pieces are permanently bonded without the assistance of any intermediate layers or field. Because there is no introduction of intermediate layers, sapphire direct bonding can provide high-temperature stable bonds [14], and at the same time avoid the problems caused by the mismatch of thermal expansion coefficients in high temperature environments. In this paper, we propose a sapphire direct bonding method based on plasma surface activation, hydrophilic pre-bonding, and high temperature annealing, and through this process the vacuum-sealed cavity targeted for high temperature applications is prepared. Ultimately, the manufactured vacuum-sealed cavity will be used for the construction of an all-sapphire pressure sensor based on extrinsic Fabry–Perot interferometer (EFPI) schematically shown in Figure 1.

## 2. Experiment

**Figure 2:**
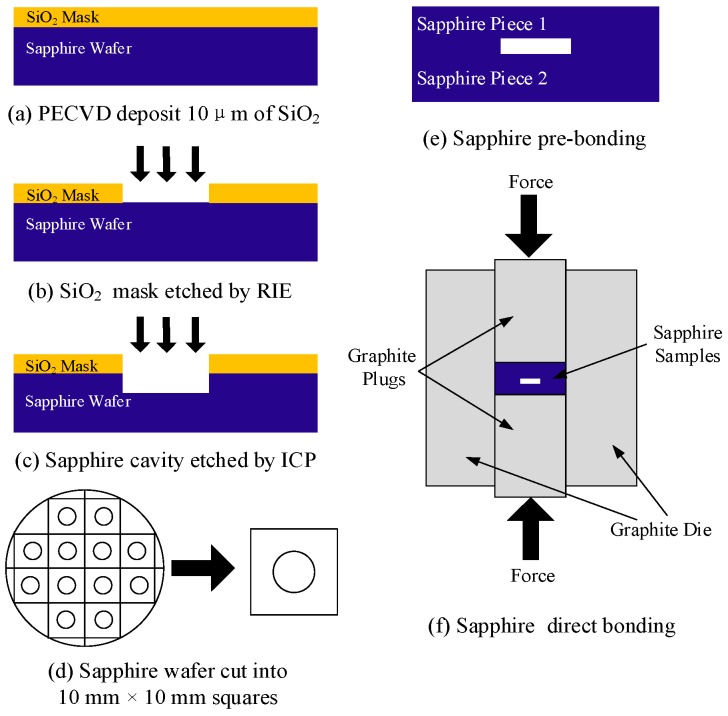
Fabrication flow chart of the vacuum-sealed cavity.

C-plane (0001) highly polished sapphire wafers, obtained commercially at low cost, were used in this experiment. The thickness of the sapphire wafers was 430 μm and the root mean square roughness was approximately 0.45 nm measured by atomic force microscopy. The reduction of the surface roughness and warpage of the wafer is critical to the realization of the direct bonding process. Therefore, the sapphire wafer was cut into 10 mm × 10 mm square pieces prior to direct bonding. The fabrication process of the vacuum-sealed cavity formed by sapphire direct bonding mainly consists of three basic steps: sapphire etching, sapphire pre-bonding, and direct bonding, as illustrated in Figure 2.

### 2.1. Sapphire Etching

The process flow of sapphire etching, as shown in Figure 2, begins with the sapphire wafer covered with a SiO_2_ film with a thickness of 10 μm. The SiO_2_ film was deposited by inductively coupled plasma-enhanced chemical vapor deposition (ICPECVD) and functioned as a mask layer. After that, the SiO_2_ mask layer was photo-patterned into 4 mm diameter circulars according to the previously designed layout, and etched by reactive ion etching (RIE), as shown in Figure 2b. Subsequently, the etching of sapphire was carried out in an Oxford inductively coupled plasma (ICP) etching system. In the experiment of sapphire etching, the physical sputter etching process, which required high energy ions to bombard the surface of the material, was employed since sapphire has excellent resistance to chemical corrosion. The etching parameters of sapphire are listed in Table 1. Under these conditions, the etching rate of sapphire reached 160 nm per minute, after 30 min, a cylindrical cavity with a depth of 4.8 μm in the sapphire wafer was obtained, and the root mean square roughness of the bottom of the etched cavity was approximately 1.63 nm, as measured by atomic force microscopy.

### 2.2. Surface Treatments and Pre-Bonding

Surface treatments of sapphire wafers are very critical to the success of direct bonding, and will directly affect the bonding interface quality of the bonded structure. The impurities adsorbed on the surface of sapphire wafers in addition to wafer warp may eventually lead to the formation of bonding voids. Moreover, the mechanical and electrical characteristics of the bonding wafers will be affected. The surface treatments of the sapphire pieces consist of two steps, namely chemical wet cleaning and plasma activation, followed by pre-bonding contact as quickly as possible.

The chemical wet cleaning of the sapphire pieces was carried out according to the RCA standard cleaning method. Firstly, the sapphire pieces were immersed in piranhas and heated to 120 °C for 30 min in order to remove the organic and metallic impurities on the surface, followed by immersion in a mixed solution of NH_4_OH, H_2_O_2_, and H_2_O to remove the particles. At the final stage of cleaning, the hydrophilic treatment of the sapphire pieces was conducted by immersing in a mixture of H_2_SO_4_ and H_2_O_2_. Finally, the pieces were rinsed with ultra pure deionized water and dried under a stream of nitrogen.

Before pre-bonding, the cleaned sapphire pieces were introduced to the PVA Tepla IoN40 plasma system and the surfaces were activated by oxygen plasma sputtering. The parameters in this activation process were as follows: power of 200 W, chamber pressure of 200 mtorr, and activation duration of 45 s. After these surface treatments, the sapphire pieces with active surfaces were brought as quickly as possible into face-to-face contact in methanol with a higher OH^−^ concentration. When the surfaces of the two sapphire pieces were in contact with each other, it could be felt that there was a faint attraction formed by van der Waals interactions or hydrogen bridge bonds in the bonding interface [15]. Subsequently, a small amount of pressure was applied to the center of the pieces to extrude the air remaining at the bonding interface, and by doing so, the bonding gaps and voids caused by multi-point contact could be avoided. The pre-bonding process of the sapphire pieces was completed at room temperature and the entire process was carried out in a class-1000 clean room. Ultimately, to strengthen the bonding, the pre-bonded sapphire pieces were transferred from the clean room to a hot press furnace for direct bonding.

### 2.3. Sapphire Direct Bonding

**Figure 3:**
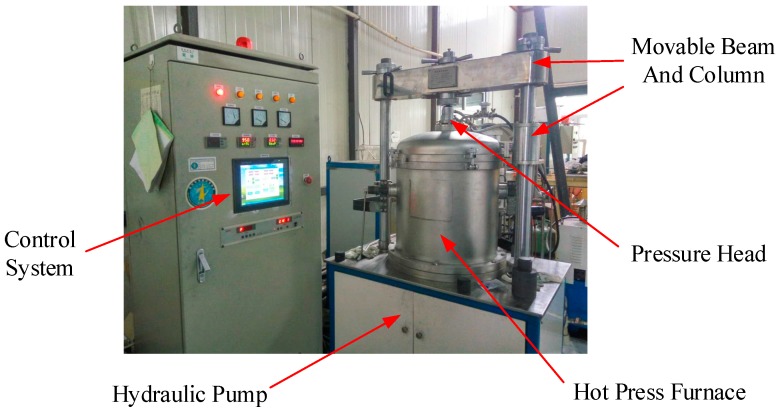
Image of the vacuum hot press furnace.

A vacuum hot press furnace, as shown in Figure 3, was used for the direct bonding of the sapphire pieces. It was also used for pressure sintering of various ceramic materials and nanomaterials. The high temperature furnace apparatus rapidly heats the sapphire pieces to be bonded via electrode heating, and adjusts the heating rate by controlling the power. The pressure is applied to the pieces by means of a hydraulic pump and a pressure head displacement control system. At the same time, the workbench and the movable beam of the hot press furnace are provided with a column connection to ensure the verticality of the applied pressure. Under high-temperature environments, the thermal expansion of the residual gas trapped in the cavity can lead to the deformation of the thin diaphragm [14]. One of the advantages of this hot press furnace is its ability to provide a vacuum environment for sapphire direct bonding, thereby forming a vacuum-sealed cavity for pressure measurement at high temperatures. In the future, the vacuum-sealed cavity will be able to eliminate the loss of the optical signal resulting from the refractive index of the trapped air when applied in fiber optic pressure sensors. 

**Figure 4:**
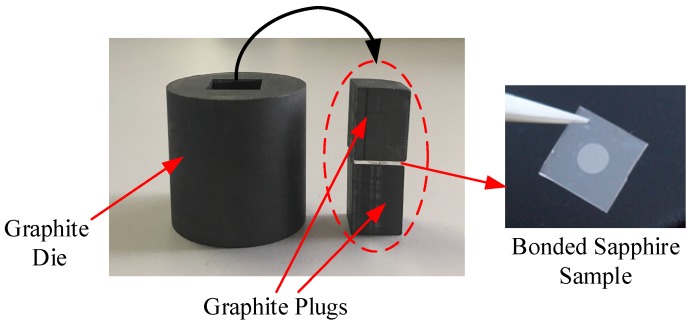
Photograph of the 30 mm diameter graphite die and bonded sapphire sample.

**Figure 5:**
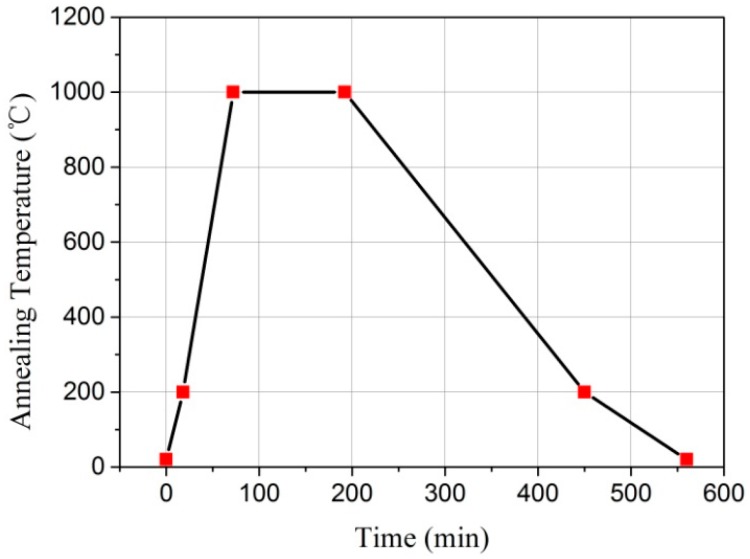
Sapphire direct bonding annealing curve.

The bonding experiment was performed using two 10 mm × 10 mm × 430 μm sapphire pieces, one of which was etched with a shallow cylindrical cavity with a diameter of 4 mm and a depth of 4.8 μm. The minimum pressure load applied to the pre-bonded sapphire pieces was limited to 14.6 MPa due to the fact that the custom furnace had a large initial pressure, so the sapphire piece with the cavity was not thinned before bonding to prevent cracking. In order to apply the pressure more evenly to the surface of the sample during the bonding process, the pre-bonded sapphire pieces were placed in a graphite die with a diameter of 30 mm and a height of 30 mm, shown in Figure 4, and then loaded into the hot press furnace for direct bonding. Sapphire direct bonding was achieved at a pressure of 14.6 MPa and a temperature of 1000 °C, for the duration of 2 h. The direct bonding annealing temperature curve of the pre-bonded sapphire pieces is shown in Figure 5, the temperature was raised from room temperature to 200 °C at a heating rate of 10 °C/min and then heated to 1000 °C at a heating rate of 15–20 °C/min. The sapphire pieces were held for 2 h at this temperature and then cooled to room temperature by means of a water cooling system. The temperature sensor was used to detect the entire temperature change process as well as display it on the screen of the control system. Inspection of the sample after direct bonding revealed no cracks, and all contacted areas of two sapphire pieces were well bonded together, as illustrated in Figure 4.

## 3. Results and Discussion

### 3.1. Leak Testing

**Figure 6:**
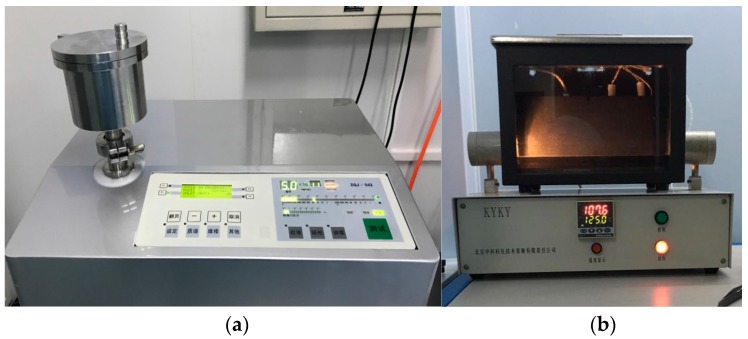
Leak testing apparatus: (**a**) helium mass spectrometer for the fine leak test; (**b**) fluorine oil leak detection device for the coarse leak test.

In order to demonstrate the tightness of the bonded cavity, a fine leak test utilizing helium mass spectrometry and a coarse leak test utilizing fluorine oil were carried out. First, the helium mass spectrometer was used to detect the leak rate of the sample, which evaluated the leak rate of the vacuum chamber by detecting the corresponding relationship between the helium content and time. According to the volume of the bonded cavity, the sample was introduced into the helium pressure chamber for 2 h at a pressure of 517 kPa, and then got removed quickly into the helium mass spectrometer for leak detection, as illustrated in Figure 6a. The test result showed that the helium leak rate of the cavity is 2.2 × 10^−9^ Pa·m^3^/s, which was less than the rejection limit of the measured leakage rate of 5 × 10^−9^ Pa·m^3^/s. In order to further eliminate the measurement error caused by the large leaks, the sample with fine leak detection by helium mass spectrometry was again tested by the fluorine oil method, as shown in Figure 6b. The sample was immersed in light fluorine oil and pressurized with nitrogen for 2 h at a pressure of 517 kPa. After that, the sample was removed from the chamber and immersed into the heavy fluorine oil. The result showed that no bubbles emerged, which indicated that the cavity was well sealed.

### 3.2. Tensile Strength Testing

**Figure 7:**
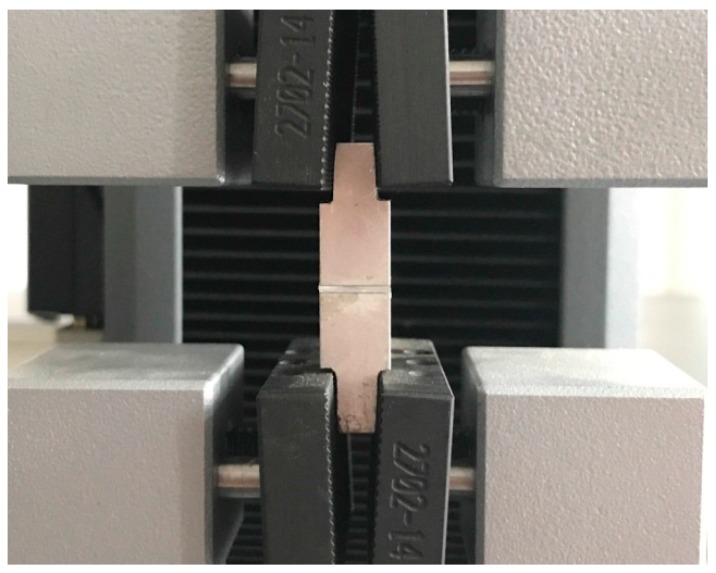
Bonded sapphire sample loaded into the tensile testing machine.

In order to evaluate the bonding strength of the sapphire bonding interface, tensile testing was performed. The bonded sample was fixed on the vertical fixture with an adhesive, as shown in Figure 7, and loaded into the Instron 2710 tensile testing machine with a load limit of 50 kN. The adhesive interface was pulled off under the load of 720 N and the bonding interface remained intact, indicating that the tensile strength of the bond interface exceeded 7.2 MPa. This value is in excess of the recommended 4–5 MPa minimum bond strength required for the manufacture of Micro-Electro-Mechanical-System (MEMS) devices [11].

### 3.3. Cross-Sectional Microstructure Observation

**Figure 8:**
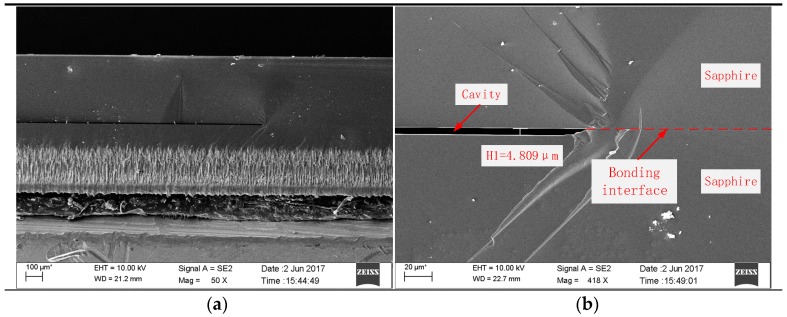
SEM images of the bonded sapphire sample: (**a**) cross-sectional image of the sapphire bonding interface; (**b**) image of the bonded cavity with a height of 4.8 μm.

In order to observe the internal bonding quality, the bonded sapphire sample was cut along the cross-section. The ultraviolet laser processing equipment was used to cut the sample to a designated depth along the cavity and then to force the sample to break along the cutting groove. Visual inspection of the exposed bonding interface was clean. After that, the cross-section of the sample was coated with a thin gold film and imaged under a scanning electron microscope (SEM). Figure 8a shows the cross-section of the sapphire bonding interface, which clearly shows the roughness introduced by laser cutting. Figure 8b shows that the cavity formed by direct bonding still maintains at a height of about 4.8 μm, the cavity structure is intact, and the bonding interface is clearly visible. 

**Figure 9:**
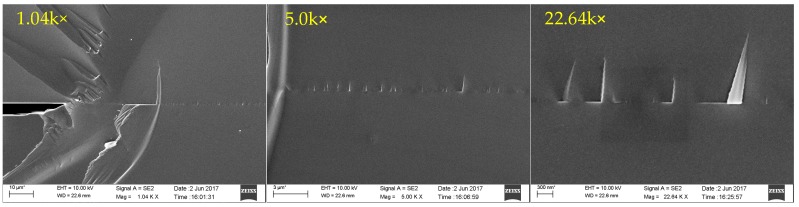
SEM images of the cross-section of the bonding interface at different magnifications.

The SEM images of the sapphire direct bonding interface at magnification levels of 1.04 k×, 5.0 k×, and 22.64 k× are shown in Figure 9. High magnification SEM research reveals that most of the bonding interface is smooth and free of voids, indicating that good bonding quality is achieved through the direct bonding process. At 22.64 k× magnification, 300-nm-scale folds formed at the break are visible on the bonding interface, which is a result of the two sapphire crystal orientations not being perfectly aligned during the bonding process.

**Figure 10:**
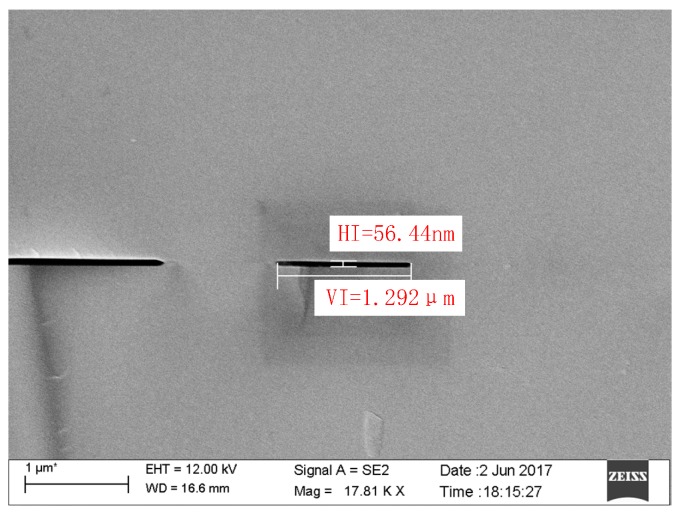
High magnification SEM image of bubble voids appearing on the bonding interface.

By scanning the entire bonding interface at high magnification, we can see that most of the interface is smooth. Only two bonding voids less than 100 nm in height were found, as shown in Figure 10. These were most likely caused by an air bubble surrounded by the interface and not vented during the bonding process. The bonding areas next to the voids, clearly visible in the high magnification SEM image, are smooth and tight.

**Figure 11:**
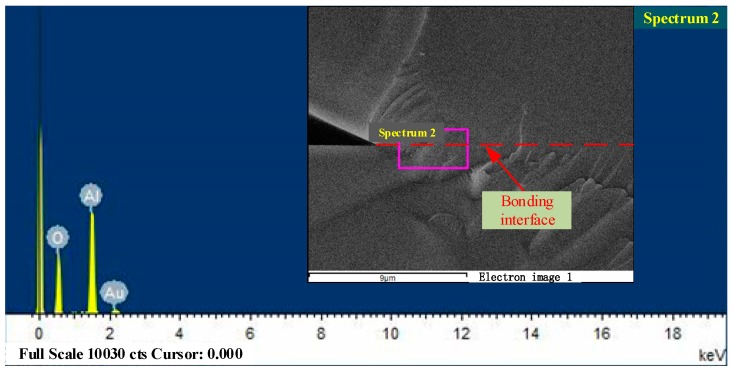
The Energy Dispersive Spectrometry (EDS) analysis results of the bonding interface.

The qualitative analysis of the bonding interface elements by the Energy Dispersive Spectrometer (EDS) attached to the electron microscope was carried out. The result shown in Figure 11 indicates that there was no formation of new elements in the bonding interface, except for the presence of the sputtered gold element.

## 4. Conclusions

In this paper, the construction of a vacuum-sealed cavity that can be used for high temperature pressure measurements was demonstrated by sapphire inductively coupled plasma etching and sapphire direct bonding processes. Sapphire direct bonding was accomplished successfully by the combination of plasma surface activation, pre-bonding, and high-temperature annealing. The SEM inspection of the bonding interface demonstrated good bond quality and the intact cavity structure. The tensile strength testing indicated that the tensile strength of the bonding interface exceeded 7.2 MPa. In addition to optic-fiber pressure sensors mentioned in this article, the cavity structure formed by sapphire direct bonding also can be extended to manufacture other MEMS devices that can potentially operate in high temperature environments. Future work may include two parts. The first one deals with the etching of deeper sensing cavities for accurate testing. The second one involves the no-glue connection of optic fiber to the sensor that could be carried out for extremely high temperature applications.

## Figures and Tables

**Table 1:** Etching parameters of sapphire.

Etching Parameters	Value
Etching equipment type	Oxford Inductively Coupled Plasma (ICP) 180
Etching gas	Mixture of BCl_3_ and Cl_2_
Gas flow	BCl_3_ 80 sccm, Cl_2_ 20 sccm
Etching power	Radio Frequency 400W, ICP 2500W
Pressure	12 mTorr
